# A bibliometric systematic review of extracellular vesicles in eye diseases from 2003 to 2022

**DOI:** 10.1097/MD.0000000000034831

**Published:** 2023-08-18

**Authors:** Xianke Luo, Xiaoling Yan, Dan Yin, Yanting Xia, Shimeng Li, Suisui shi, Miaoran Gao, Changlu Yang, Jian Zhou

**Affiliations:** a Dongfang Hospital, Beijing University of Chinese Medicine, Beijing, China; b Dongzhimen Hospital, Beijing University of Chinese Medicine, Beijing, China.

**Keywords:** aqueous humor, bibliometric, exosomes, extracellular vesicles, eye diseases, mesenchymal stem cell

## Abstract

**Background:**

Extracellular vesicles (EVs) have emerged as a valuable and promising research field in eye diseases. However, there are few bibliometric studies in this area. The purpose of this study was to employ bibliometric analysis to visualize the research hotspots and trends of EVs in eye diseases and provide researchers with new perspectives for further studies.

**Methods:**

Articles and reviews on EVs in eye diseases published between January 1, 2003 and December 31, 2022 were retrieved from the Web of Science Core Collection. Qualitative and quantitative analysis was performed using Microsoft Excel and CiteSpace software.

**Results:**

In total, 790 articles were included in the analysis. Over the past 2 decades, there has been a significant increase in the number of publications on the study of EVs in eye diseases. The United States, China, and Italy made the most significant contributions to this field. The Chinese Academy of Sciences was the most productive institution, and *International Journal of Molecular Sciences* published the most number of articles. *Proceedings of the National Academy of Sciences of the United States of America* had the highest citation frequency. Beit-Yannai E had the highest output and Thery C had the highest average citation frequency among authors. The analysis of keywords revealed that the neuroprotective effects of stem cell-derived EVs and biomarkers of eye diseases are current research hotspots and frontiers in this field.

**Conclusion:**

This study provides a scientific perspective on EVs in eye diseases and provides valuable information for researchers to detect current research conditions, hotspots, and emerging trends for further study.

## 1. Introduction

Extracellular vesicles (EVs) are a hypernym for a heterogeneous group of phospholipid bilayer-encased membranous nanovesicles that can be classified into 3 major categories based on their biogenetic pathway, composition, and physical characteristics: apoptotic bodies (50–2000 nm), microvesicles (50–1500 nm), and exosomes (50–120 nm).^[1]^ EVs contain mRNA, miRNA, lipids, and proteins that can be delivered to nearby or distant cells via blood flow, making them a promising tool for intercellular communication, disease progression, biomarker discovery, and potential therapeutic applications. Recent studies have explored the potential of EVs as an alternative to paracrine-acting cellular therapies, including those for eye-related conditions.

Bai et al^[2]^ demonstrated that exosomes derived from human umbilical cord mesenchymal stem cells (MSC) significantly reduced the severity of experimental autoimmune uveitis by decreasing the infiltration of T-cell subsets and other inflammatory cells in the eyes. Yu et al^[3]^ showed that exosomes derived from human umbilical cord MSC improve laser-induced retinal damage by downregulating MCP-1 expression. Furthermore, the biomolecular composition of EVs varies depending on their physiological state and cell of origin, making them attractive biomarkers for various diseases.^[4]^ Kang et al^[5]^ identified changes in EVs proteins present in ARPE-19 cell culture supernatants and aqueous humor from individuals with neovascular age-related macular degeneration compared with unaffected individuals. Therefore, exosomal proteins in the aqueous humor may serve as potential biomarkers for age-related macular degeneration.

Bibliometrics is an interdisciplinary field that utilizes mathematical and statistical methods to evaluate and track the advancement of specific disciplines by analyzing published data.^[6,7]^ Through bibliometric analysis, it is possible to estimate the outputs and citations of countries, institutions, and authors. Additionally, the frequency of research keywords related to hotspots and frontiers in particular fields also can be determined.^[8]^ Bibliometric analysis has been widely used in medical fields, such as gynecology, orthopedics, and complementary and alternative medicine, promoting the development of these fields.^[9–11]^ However, there is currently a lack of bibliometric studies on the role of EVs in eye diseases. Therefore, the purpose of this study was to systematically analyze the research on EVs in eye diseases and evaluate the current state and hotspots in this field.

## 2. Methods

### 2.1. Data collection and search strategy

**Figure 1.**
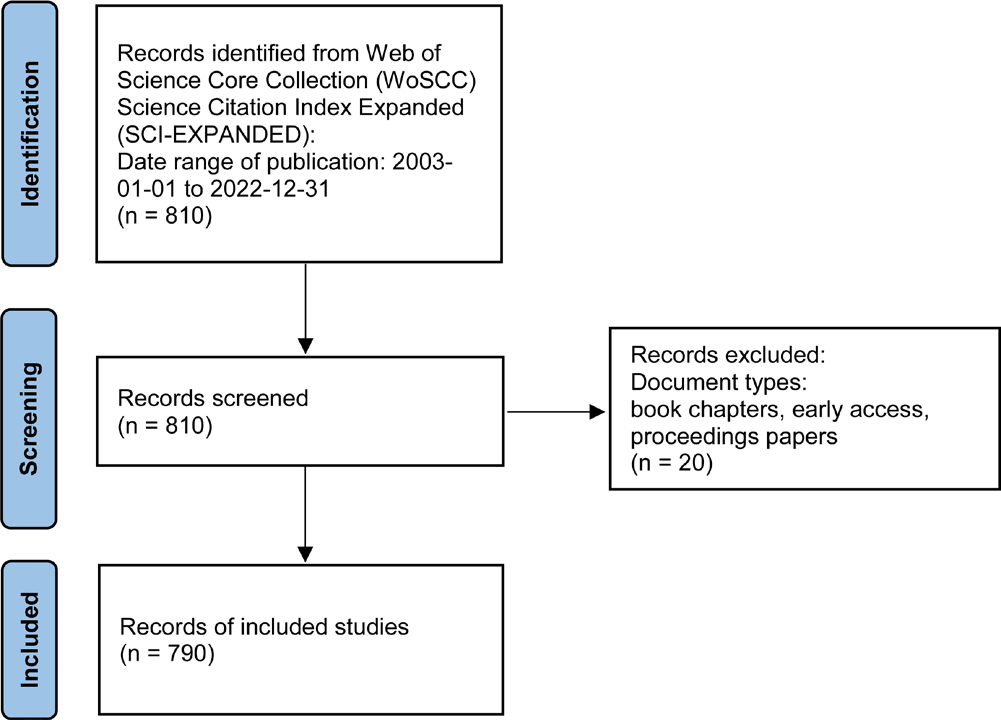
Study data retrieval flow chat.

The Web of Science (WoS) is widely recognized as one of the most comprehensive and authoritative database platforms for obtaining global academic information, with over 12,000 international academic journals included in its database.^[12]^ For this study, the WoS core collection database was chosen as the literature source, covering the period from January 1, 2003, to December 31, 2022. The search strategy was as follows: TS = (extracellular vesicle* OR exosome* OR cell-derived microparticle*) AND TS = (eye* OR oculopathy OR optic* OR ophthalmic* OR ophthalmo* OR ocular). The search was limited to English articles and reviews, while early access, proceedings papers, and book chapters were excluded. Ultimately, a total of 790 valid documents were obtained and exported in the form of “full record and cited references” for further analysis. As all the data used in this study were obtained from public databases, no ethics committee approval or informed consent was required. The search process was carried out by 2 individuals independently, and in case of any disagreement, a third experienced corresponding author was consulted to resolve the issue before making the final decision. The screening flow is illustrated in Figure 1.

### 2.2. Analysis tool

CiteSpace is a Java application developed by Dr Chaomei Chen in 2004. This interactive visualization tool combines information visualization methods, bibliometrics, and data-mining algorithms to provide scientific and intuitive support for clinicians and researchers in a given field. CiteSpace, designed specifically for bibliometric analysis, integrates analysis functions for countries/regions, authors, institutions, journals, citations, and keyword information, making it a widely used tool in academic research to identify research frontiers, hotspots, and trends.

### 2.3. Data analysis

A chart of the annual publications was generated using Microsoft Excel 2021. The data were then imported into the CiteSpace (6.1. R6), the year per slice was 1 year, and the nodes included in the analysis were author, institution, country/region, keyword, reference, cited author, and cited journal. The g-index (k = 25) was selected in the selection criteria, and the pathfinder and pruning sliced networks were selected in the pruning module. The other parameter settings were based on the initial software settings.

## 3. Results

### 3.1. Annual publication outputs and trends

**Figure 2.**
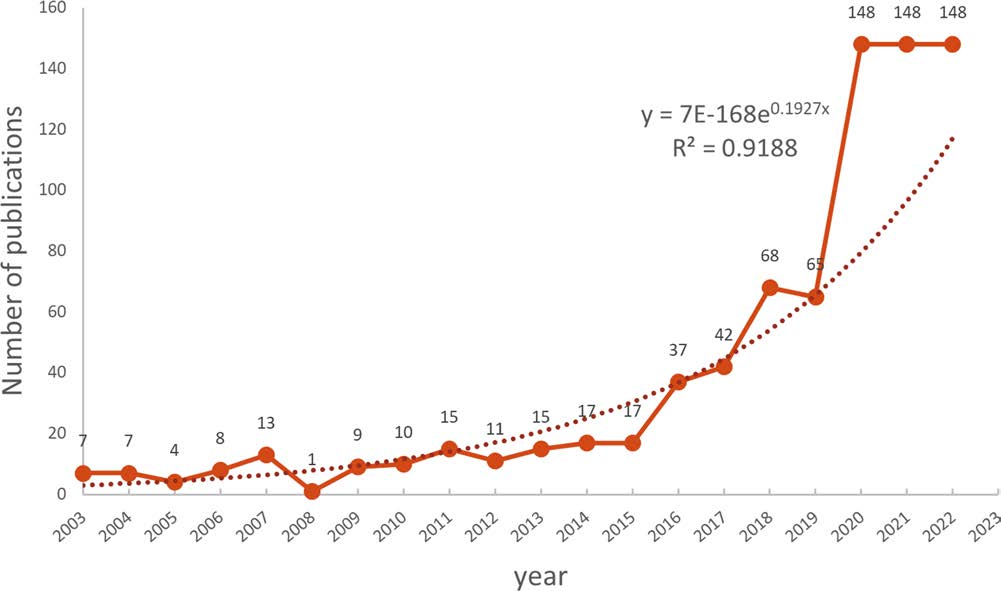
Annual number of publications worldwide from 2003 to 2022. The fitting formula was y = 7E-168e0.1927x.

Between January 1, 2003 and December 31, 2022, the WoS published a total of 790 publications related to EVs in eye diseases, comprising 612 research articles and 178 reviews. Figure 2 illustrates a distinct 3-stage research trend. The first stage, spanning from 2003 to 2015, saw a relatively stable number of publications, ranging from 1 article in 2008 to 17 articles in 2015, with a total of 183 publications. The second stage, from 2016 to 2019, was characterized by a remarkable surge in published articles. Finally, from 2020 onwards, a sudden upward trend emerged, with 148 articles published in 2020, more than double that of the previous year. Based on a fitting curve (y = 7E-168e0.1927x) with R2 = 0.9188, as shown in Figure 2, it was possible to forecast the potential increase in annual publication outputs.

### 3.2. Analysis of countries

**Figure 3.**
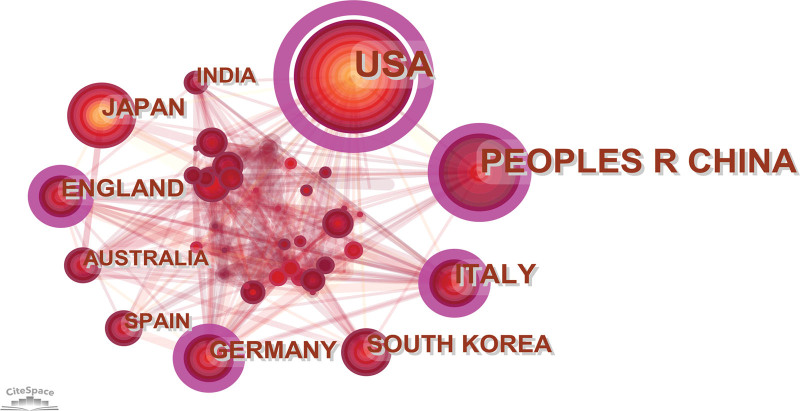
National cooperation visualization map. The cooperation network map of countries related to EVs in eye diseases from 2003–2022. Node size represents the number of output. The thicker the pink outer ring of the node, the higher the centrality. At the same time, the connecting line between nodes indicates the collaborative relationship between countries. EVs = extracellular vesicles.

In the field of EVs in eye diseases, 790 records have been published across 67 countries/regions on 5 continents. The United States had the highest number of publications, contributing 287 articles, followed closely by China with 158 articles, and Italy with 60 articles (Table 1). We also created a cooperation network diagram (Fig. 3) to illustrate the relationships among different countries or regions. Each colored ring node represents a country, with a size corresponding to the number of publications. The thickness and number of lines between nodes indicate the degree of cooperation, while the color bandwidth represents the number of publications for the current year, with wider ribbons indicating a greater number of published articles. A purple-ringed circle represents a pivotal point within the field, indicating a higher centrality. Countries such as the United States, China, Italy, England, and Germany are circled in purple, highlighting their significance in connecting the field.

**Table 1:** Top 10 prolific countries/regions and institutions.

Rank	Country/region	Count	Centrality	Institution	Count	Centrality
1	USA	287 (36.33%)	0.74	Chinese Acad Sci (China)	16 (3.98%)	0.06
2	China	158 (20.00%)	0.18	Harvard Univ (USA)	15 (3.73%)	0.1
3	Italy	60 (7.59%)	0.1	Duke Univ (USA)	14 (3.48%)	0.03
4	South Korea	51 (6.46%)	0.07	Shanghai Jiao Tong Univ (China)	13 (3.23%)	0.02
5	England	42 (5.32%)	0.15	Univ Amsterdam (Holland)	12 (2.99%)	0.1
6	Japan	38 (4.81%)	0.02	Seoul Natl Univ (South Korea)	12 (2.99%)	0
7	Germany	35 (4.43%)	0.11	Univ Illinois (USA)	11 (2.74%)	0.1
8	Australia	31 (3.92%)	0.08	Southeast Univ (China)	10 (2.49%)	0.04
9	Spain	31 (3.92%)	0.07	Johns Hopkins Univ (USA)	9 (2.24%)	0
10	India	30 (3.80%)	0.05	Tianjin Med Univ (China)	9 (2.24%)	0

### 3.3. Analysis of institutions

**Figure 4.**
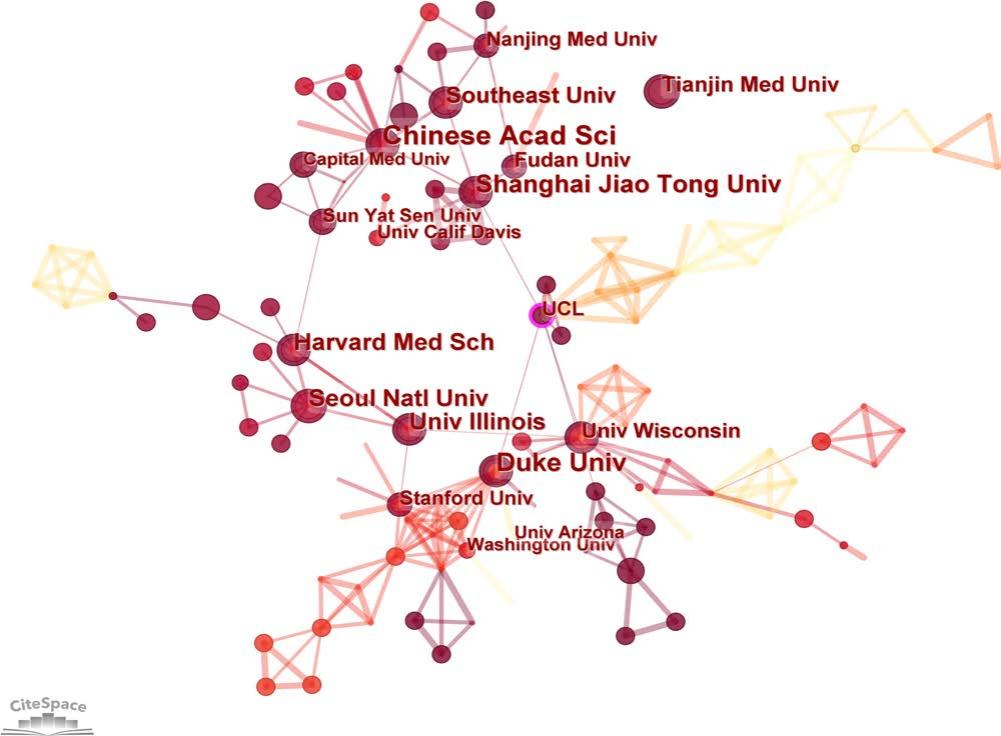
Institutional cooperation visualization map. The cooperation network map of productive authors related to EVs in eye diseases from 2003–2022. Each node indicates an institution, and different colors in the nodes indicate different publication years. At the same time, the connecting line between nodes indicates the collaborative relationship between institutions. EVs = extracellular vesicles.

Analyzing research institutions can provide valuable insights into the global distribution of research on EVs in eye diseases, enabling scholars to identify potential partners and foster collaboration. In total, 790 qualified articles were published by 405 institutions. As shown in Table 1, the Chinese Academy of Sciences emerged as the leading institution in terms of publications, followed by Harvard University and Duke University. Among the top 10 universities, 8 were from the United States and China. Figure 4 illustrates that key institutional partnerships mainly focus on domestic cooperation, as exemplified by the close ties between the Chinese Academy of Sciences, Shanghai Jiao Tong University, and Southeast University. However, there are few institutional links between countries. Notably, University College London, highlighted in purple, is not among the top 10 prolific institutions, but exhibits the strongest centrality and therefore plays an important role as a bridge in the field.

### 3.4. Analysis of journal

Scholarly articles on EVs in eye diseases have been published in 638 journals. Table 2 provides a list of the top 10 most productive and co-cited journals, along with their impact factor (IF) and Journal Citation Reports partition. *The International Journal of Molecular Sciences* had the highest number of outputs (28, 3.54%), followed by Experimental Eye Research (22, 2.78%), Scientific Reports (20, 2.53%), and Investigative Ophthalmology & Visual Science (17, 2.15%). Among the top 10 journals, Nature Communications had the highest IF (17.694), followed by the Journal of Extracellular Vesicles with an IF of 17.337. Analyzing the distribution of published articles across sources can help identify core journals.

**Table 2:** Top 10 journals and co-cited journals.

Rank	Journal	Count (%)	IF (2021)	JCR	Co-cited journal	Count	IF (2021)	JCR
1	*International Journal of Molecular Sciences*	28 (3.54%)	6.208	Q1	*Proceedings of the National Academy of Sciences of the United States of America*	420	12.779	Q1
2	*Experimental Eye Research*	22 (2.78%)	3.77	Q2	*Sci Rep-UK*	409	4.997	Q2
3	*Scientific Reports*	20 (2.53%)	4.997	Q2	*Plos One*	385	3.752	Q2
4	*Investigative Ophthalmology and Visual Science*	17 (2.15%)	4.925	Q1	*Nature*	342	69.504	Q1
5	*Stem Cell Research and Therapy*	17 (2.15%)	8.088	Q1	*Science*	324	63.832	Q1
6	*Analytical Chemistry*	16 (2.03%)	8.008	Q1	*Nature Communications*	288	17.694	Q1
7	*Cells*	14 (1.77%)	7.666	Q2	*Journal of Biological Chemistry*	287	5.485	Q2
8	*Nature Communications*	12 (1.52%)	17.694	Q1	*Journal of Extracellular Vesicles*	285	17.337	Q1
9	*Journal of Extracellular Vesicles*	11 (1.39%)	17.337	Q1	*Investigative Ophthalmology and Visual Science*	262	4.925	Q1
10	*Plos One*	11 (1.39%)	3.752	Q2	*Cell*	244	66.85	Q1

IF = impact factor, JCR = journal citation reports.

Co-citation analysis measures the degree of the relationship between articles and reflects the influence of journals in specific research fields. As shown in Table 2, Proceedings of the National Academy of Sciences of the United States of America was the most frequently cited journal (420), followed by Scientific Reports (409), and Plos One (409). Among the top 10 journals, Nature had the highest IF (69.504), followed by Cell, with an IF of 66.85. According to the Journal Citation Reports partition analysis, 7 of the top 10 most productive journals were classified as Q1, while 6 of the top 10 co-cited journals were classified as Q1.

**Figure 5.**
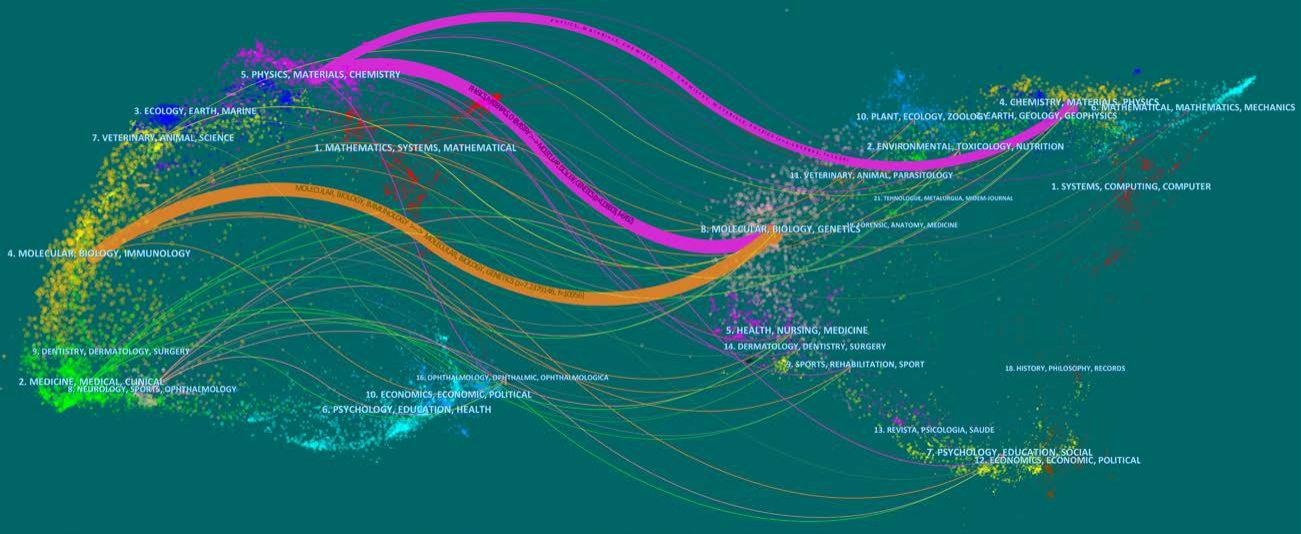
Dual-map overlay of journals. The left nodes represent the included literature; the right represent the references in the literature. The labels represent the discipline. The link represents the cited path.

The overlay of dual-map for journals depicts the distribution of the relationships between journals. Citing journals are situated on the left, while cited journals are located on the right. Colored paths are used to indicate the cited relationships. In Figure 5, the orange path indicates that articles published in Molecular/Biology/Immunology journals are frequently cited by Molecular/Biology/Genetics journals. Likewise, the purple path suggests that articles published in Physics/Materials/Chemistry journals are commonly cited by Molecular/Biology/Genetics and Chemistry/Materials/Physics journals.

### 3.5. Analysis of authors and cited authors

**Figure 6.**
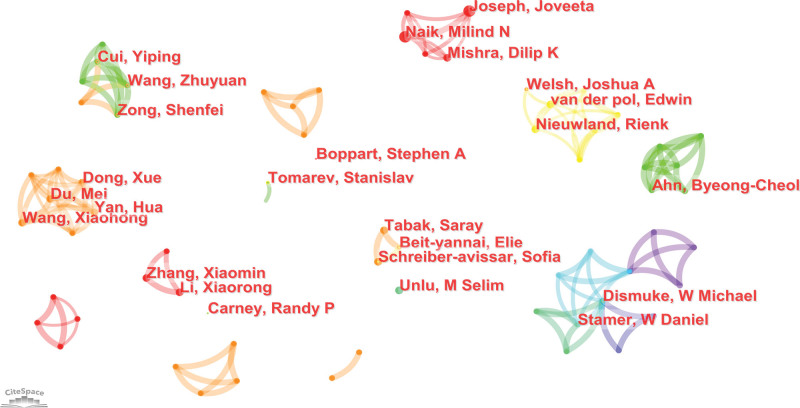
Author cooperation visualization map. The cooperation network map of productive authors related to EVs in eye diseases from 2003–2022. Each node indicates an author, and different colors in the nodes indicate different publication years. At the same time, the connecting line between nodes indicates the collaborative relationship between authors. EVs = extracellular vesicles.

Our study included 575 authors and 813 co-cited authors. As the nodes of the authors are scattered on the map, this study shows a part of the whole map concentrated distribution (Fig. 6). The top 3 authors in terms of publications were Beit-Yannai E (6, 1.04%), Schreiber-Avissar S (5, 0.87%), and Dismuke WM (5, 0.87%) (Table 3). Despite the strong collaboration observed within each research team, all authors showed low centrality values, indicating limited collaboration among the researchers. The author collaboration network depicted in Figure 6 further highlights the lack of interteam collaboration in this field. Among the 813 co-cited authors, Thery C had the highest co-citation count and centrality, while Valadi H and Im H also showed high centrality (≥0.1).

**Table 3:** Top 10 productive authors and co-cited authors.

Rank	Author	Publication	Centrality	Rank	Co-cited author	Citation	Centrality
1	Beit-Yannai E	6 (1.04%)	0	1	Thery C	192	0.23
2	Schreiber-Avissar S	5 (0.87%)	0	2	Raposo G	92	0.08
3	Dismuke WM	5 (0.87%)	0	3	van der Pol E	81	0.05
4	Zong S	5 (0.70%)	0	4	Valadi H	78	0.1
5	Stamer WD	4 (0.70%)	0	5	Im H	69	0.11
6	Wang Z	4 (0.70%)	0	6	Colombo M	67	0.03
7	Naik MN	4 (0.70%)	0	7	Mead B	59	0.04
8	Tomarev S	4 (0.70%)	0	8	Zhang Y	58	0.01
9	Cui Y	4 (0.70%)	0	9	Kalluri R	52	0.01
10	Joseph J	4 (1.04%)	0	10	van Niel G	52	0.02

### 3.6. Analysis of reference

When 2 or more references are cited in multiple articles, they are considered to have a co-citation relationship. The top 10 most co-cited references among the 789 cited references, which have an important role in the field, are shown in Table 4. These highly co-cited articles are highly recommended for researchers seeking to conduct rigorous research.

**Table 4:** Top 10 co-cited references.

Rank	Author	Year	Reference	Co-citations	Centrality
1	Thery C	2018	Minimal information for studies of extracellular vesicles 2018 (MISEV2018): a position statement of the international society for extracellular vesicles and update of the MISEV2014 guidelines	54	0.02
2	van Niel G	2018	Shedding light on the cell biology of extracellular vesicles	45	0.01
3	Mead B	2017	Bone Marrow-derived mesenchymal stem cells-derived exosomes promote survival of retinal ganglion cells through miRNA-dependent mechanisms	39	0
4	Kalluri R	2020	The biology, function, and biomedical applications of exosomes	37	0
5	Samaeekia R	2018	Effect of HUMAN CORNEAL MESENCHYMAL STROMAL Cell-derived exosomes on corneal epithelial wound healing	36	0.01
6	Im H	2014	Label-free detection and molecular profiling of exosomes with a nano-plasmonic sensor	36	0.1
7	Bai LL	2017	Effects of mesenchymal stem cell-derived exosomes on experimental autoimmune uveitis	33	0.08
8	Mathieu M	2019	Specificities of secretion and uptake of exosomes and other extracellular vesicles for cell-to-cell communication	29	0
9	Klingeborn M	2017	Roles of exosomes in the normal and diseased eye	27	0
10	Colombo M	2014	Biogenesis, secretion, and intercellular interactions of exosomes and other extracellular vesicles	26	0.02

### 3.7. Analysis of keyword

Keywords are essential indicators of publication topic. Analyzing the co-occurrence of keywords can reveal research hotspots and predict trends in a given field. When 2 or more keywords appear in the same literature, they are considered to co-occur. Table 5 presents the top 10 keywords ranked by co-occurrence frequency. Notably, “extracellular vesicle,” “mesenchymal stem cell,” “protein,” “microvesicle”, and “stem cell” were among the most frequent ones, indicating that they are currently the research hotspots for EVs in eye diseases.

**Table 5:** Top 10 keywords in terms of frequency.

Ranking	Keyword	Frequency	Centrality
1	Extracellular vesicle	258	0.05
2	Exosm	179	0.12
3	Mesenchymal stem cell	65	0.05
4	Protein	57	0.11
5	Microvesicle	49	0.07
6	Stem cell	45	0.04
7	In vitro	40	0.03
8	Microparticle	33	0.04
9	Extracellular matrix	33	0.19
10	Biomarker	32	0.03

**Figure 7.**
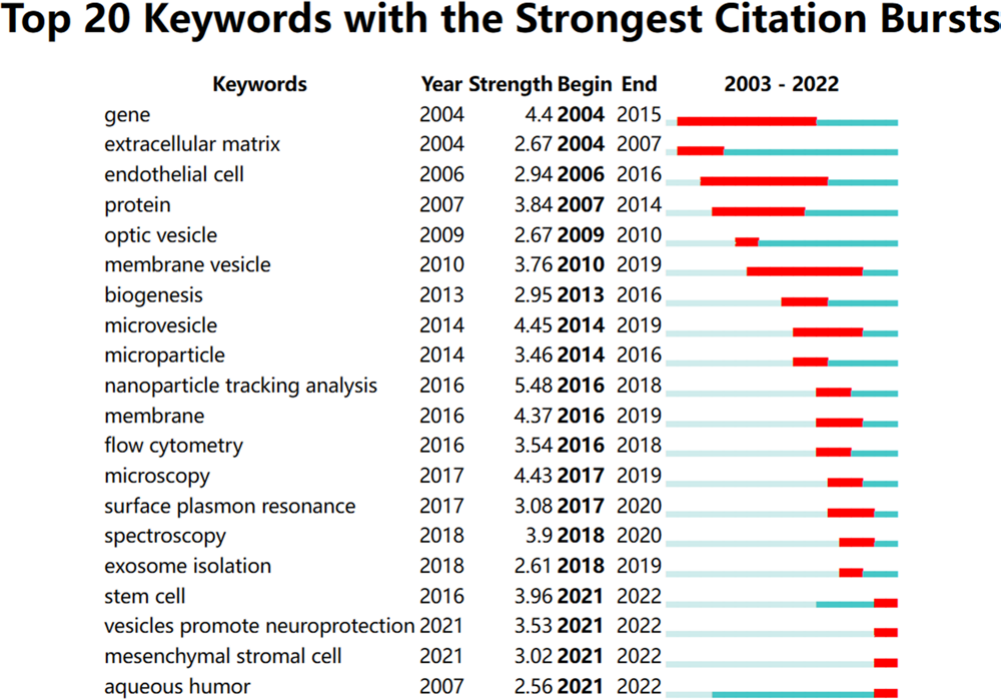
Top 20 keywords with the strongest citation bursts.

Keyword bursts are crucial indicators of the most active research topics and can predict the direction of cutting-edge research and identify potential hotspots in the field. Figure 7 illustrates the top 20 keywords with the strongest citation bursts, with a minimum duration of 1 year and γ = 0.84. From 2003 to 2014, the predominant research trends on EVs of eye diseases focused on their concept, composition, and contents, as evidenced by keywords such as “gene,” “protein,” “optic vesicle,” “membrane vesicle,” “microvesicle”, and “microparticle.” Between 2016 and 2018, the research emphasis shifted towards the development and application of detection and identification methods for EVs, including “nanoparticle tracking analysis,” “flow cytometry,” “microscopy,” “surface plasmon resonance,” “spectroscopy”, and “exosome isolation.” Recently, researchers have explored the protective effects of stem cells on nerves and the role of EVs in the aqueous humor, as indicated by keywords such as “stem cell therapy,” “vesicles promoting neuroprotection,” “mesenchymal stromal cells, and “aqueous humor.”

**Figure 8.**
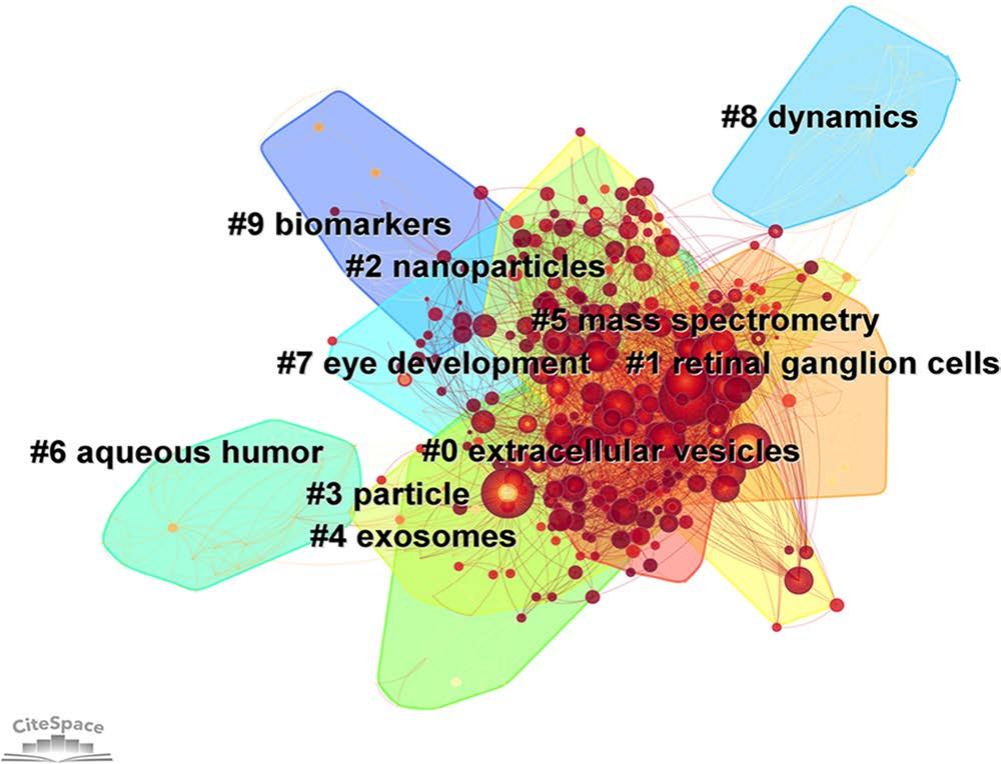
Keywords cluster map.

Keyword clusters are networks of clusters formed by keywords with similar research topics that reveal the main themes. In our study, 43 clusters were obtained using the CiteSpace software (https://citespace.podia.com/). Figure 8 displays the 10 largest clusters, ranked from largest to smallest: “extracellular vesicles” # 0, “retinal ganglion cells” # 1, “nanoparticles” # 2, “particle” # 3, “exosomes” # 4, “mass spectrometry” # 5, “aqueous humor” # 6, “eye development” # 7, “dynamics” # 8, and “biomarkers” # 9.

## 4. Discussion

### 4.1. General information

In this study, we conducted a bibliometric analysis of literature on EVs in eye disease research between 2003 and 2022. The aim was to provide a comprehensive picture of the current research status and trends in the field and to provide scholars with a systematic reference framework for understanding important results and frontier hotspots. Our findings indicate that research in this field was in its nascent stage, from 2003 to 2015, with an average of only 10.3 articles published per year. However, the number of articles grew significantly from 2016 to 2019, reaching an average of 53 articles per year. Since 2020, the number of relevant articles has shown an even more explosive growth, indicating that research on EVs in eye diseases has attracted widespread attention. By visualizing the distribution of countries and institutions, we found that the United States and China are the major countries for EVs research in eye diseases, with 8 of the top 10 research institutions located in these 2 countries. However, we observed that the collaboration network among countries was not strong, especially the intensity of collaboration among countries with more publications was low. For instance, although the United States and China have the most publications, the intensity of connections between them is low, and there is no cooperation between China and Italy. From the perspective of research institutions, we found that collaborating institutions are mostly limited to internal connections within the same country, and cross-country collaboration and the exchange of achievements are clearly insufficient. Therefore, we suggest strengthening international interinstitutional cooperation to promote the long-term development of EVs in the field of ophthalmology.

According to research results, the *International Journal of Molecular Sciences* (IF = 6.208, Q1) is the most popular journal for studies on EVs in eye diseases. *Nature Communications* (IF = 17.694, Q1) and *Journal of Extracellular Vesicles* (IF = 17.337, Q1) have the highest IF in this research area. Co-cited journals, including prestigious publications such as Nature, Science, Nature Communications, Journal of Biological Chemistry, and Cell, suggest that EVs have attracted the attention of leading scholars in eye disease research, highlighting their significance and complexity. The double-map overlay illustrates the interdisciplinary nature of this field, encompassing molecules, biology, physics, and genetics, with limited clinical studies. These findings indicate that the current research on EVs in eye diseases is largely focused on basic research. Future research should pay more attention to clinical research to explore the potential application of EVs in the treatment of eye diseases.

Beit-Yannai E from the Ben-Gurion University of the Negev is the most prolific author in the field. His research focuses on the mechanisms of signaling between non Pigmented Ciliary Epithelium-derived EVs and Trabecular Meshwork cells, with the aim of finding feasible ways to lower intraocular pressure and treat glaucoma.^[13–15]^ Among the co-cited authors, Théry C from Institut Curie, Paris, France, had the highest co-citations and centrality. In 2006, he proposed simple and reliable methods for purifying and characterizing exosomes^[16]^ which has been widely cited by researchers and has made significant contributions to the field of exosome research. In 2016, Théry C^[17]^ reported the first large, detailed survey of worldwide practices for the isolation and characterization of EVs, which has attracted widespread attention among researchers. Additionally, Valadi H and Im H had high centrality. A study by Valadi H^[4]^ showed that after transfer of mouse exosomal RNA to human mast cells, new mouse proteins were found in the recipient cells, indicating that exosomes contain mRNA, which can be delivered to another cell and can be functional in this new location. Im H and colleagues^[18]^ reported a surface plasmon resonance-based assay for label-free, high-throughput exosome protein analysis, proposing a more sensitive detection method for the quantitative analysis of EVs. However, as Figure 5 shows, most researchers are limited to internal collaborations, which may impede discipline development. Therefore, we strongly recommend introducing specialized talent or dispatching personnel from domestic or institutional organizations to advanced institutions such as the Chinese Academy of Sciences or Harvard University for related training or academic exchanges to accelerate research progress in this field.

By analyzing co-cited references, it is possible to identify foundational literature for research in the field.^[19]^ As shown in Table 4, the top-ranked literature is a position statement and guideline for studies on EVs, which was published by the International Society for EVs in 2018. It offers researchers a clear, detailed, and easy-to-follow framework to document the experimental processes related to EVs and report their findings.^[20]^ This will help improve the quality and comparability of EVs research and promote the development of this field. Moreover, the guideline provides recommendations on how to standardize the methods and experimental procedures of EVs research to ensure the quality, reproducibility, and comparability of data.

Among the other 9 highly co-cited literature, most of them were reviews that outlined the biological characteristics, molecular mechanisms, functions, and biomedical applications of EVs, as well as the roles of exosomes in intercellular communication within the eye, and how they contribute to both normal physiological processes and disease pathogenesis. Other documents included landmark studies in this field. For instance, Mead B et al^[21]^ demonstrated for the first time that bone marrow-derived MSC-derived exosomes offer significant therapeutic benefits for the protection of retinal ganglion cells (RGCs), an effect mediated by their miRNA rather than protein content. Additionally, MSC-derived exosomes have been shown to effectively ameliorated experimental autoimmune uveoretinitis by inhibiting the migration of inflammatory cells.^[2]^ Another study has demonstrated that human corneal MSC-derived exosomes can accelerate corneal epithelial wound healing.^[22]^ Notably, as seen in the top 10 co-cited references, researchers have focused more on exosome-related studies on EVs for eye diseases than on microvesicles and apoptotic bodies.

### 4.2. Hotspots and frontiers

Keywords can be used to quickly identify hotspots within a field. Excluding common keywords such as “extracellular vesicles,” “exosomes,” “microvesicle”, and “microparticle,” the frequently appearing keywords in Table 5 include “mesenchymal stem cell,” “protein,” “stem cell,” “in vitro,” “extracellular matrix”, and “biomarker.” The keywords bursts in Figure 7 show that “stem cells,” “vesicles promote neuroprotection,” “mesenchymal stem cell”, and “atrial fluid” are the frontiers themes of research in this field. Based on the results of the keyword co-occurrence analysis, keyword clustering analysis, and keyword bursts, it can be inferred that research hotspots and frontiers in the field of EVs in eye diseases have primarily concentrated on the following areas:

#### 4.2.1. Neuroprotective effects of stem cell-derived EVs.

Stem cells have the potential to differentiate and proliferate with self-renewal and replication, producing highly differentiated functional cells. MSC are a specific type of stem cells that can be obtained from various mesenchymal tissues, including umbilical cord blood,^[23]^ bone marrow,^[24]^ dental pulp^[25]^ and adipose.^[26]^ Despite the advantages of MSC transplantation, concerns regarding allogeneic and heterologous immunological rejection, malignant transformation, and obstruction of small vessels persist. Stem cells can secrete EVs, including exosomes and microvesicles, which contain various components, such as proteins, microRNAs, messenger RNAs, and cytokines, and play crucial roles in intercellular communication and regulation.^[27]^ Stem cell-derived EVs offer a new avenue for cell-free therapy in regenerative medicine, as they have effects similar to those of stem cells in tissue regeneration and can easily traverse biological barriers and enter target organs because of their nanometer size.^[28,29]^

RGCs are essential for vision as they transmit electrochemical information to the brain via their axons in the optic nerve. Dysfunction or loss of RGCs can lead to significant visual impairment and a reduced quality of life.^[30]^ Recent research has shown that EVs derived from MSC have therapeutic effects in promoting the protection and regeneration of central nervous system neurons, including RGCs.^[31]^ Mathew et al^[32]^ found that EVs could be taken up by retinal neurons, RGCs, and microglia, making them a promising therapy for neuroprotection and regeneration in retinal diseases. Specifically, the miRNA cargoes (miR-100-5p, miR-106a-5p, miR-126-5p, miR-486-5p, and miR-144-5p) of bone marrow stem cell-derived exosomes have been shown to provide significant neuroprotection to RGCs.^[30]^ However, the precise mechanism underlying the neuroprotective effects of these miRNAs on RGCs remains largely unknown, and further studies are needed to validate the long-term efficacy of exosome therapy.^[33]^ Furthermore, human embryonic stem cell-derived MSC and EVs have also been shown to promote neuroprotection and functional preservation of RGCs in optic nerve crush mice, with rescue of tauopathy process.^[34]^ These findings suggest that MSC-derived EVs hold great promise as a neuroprotective therapy for the survival and regeneration of RGCs in retinal diseases.

Although MSC-EVs have shown promising results in treating animal models of eye diseases, their clinical application has been limited by several factors. Technical and ethical issues, as well as the challenge of obtaining sufficient samples and donor sources for research, have prevented the translation of personalized EVs therapy into clinical practice. Additionally, there is currently no efficient method for isolating pure EVs in large quantities, resulting in either a poor yield of EVs or unexpected outcomes.^[35,36]^ Furthermore, issues such as treatment dosage, administration route, and immunocompromised subjects must be addressed before MSC-EVs can be used in clinical settings.

#### 4.2.2. Biomarkers for eye disease.

EVs are now widely recognized as evolutionarily conserved intercellular communication vehicles that regulate cellular proliferation, migration, organization, and phenotypes during development, maintenance and function, injury and disease, and aging.^[37]^ Hence, EV research on EV pathophysiology and the use of EVs as biomarkers and potential therapeutics has increased dramatically in recent years. Exosomes have been found to exhibit significant differences in counts and biomolecular composition between healthy individuals and those with diseases, making them a promising biomarker for low-invasive diagnosis, observation, and prediction of several diseases, including eye diseases. Dismuke et al highlighted the potential of exosomes and their RNA payload as specific biomarkers by demonstrating that exosomes are the major EVs in the aqueous humor and carry characteristic exosomal cargoes. The rich diversity of proteins and RNAs in exosomes makes them promising avenues for exploring the pathogenesis and diagnosis of eye diseases. Yao et al^[38]^ compared exosomal protein profiles in the aqueous humor of age-related macular degeneration patients treated with anti-VEGF, and observed a decrease in the amount of SERPINA1 and AZGP1 with the duration of anti-VEGF treatment. These unique proteins may serve as new targets for drug discovery or as biomarkers for efficacy assessment. Additionally, exosomes released from the basal side of the retinal pigment epithelium, the primary site of lesions during age-related macular degeneration, enter the bloodstream from the choroid and have the potential to serve as biomarkers for the diagnosis of retinal disease.^[35,39]^ In addition, a study comprehensively investigated the global proteome of EVs in a mice model of Staphylococcus aureus endophthalmitis. This study found that Annexin A5, cathepsin D, and C5a were significantly downregulated in infected mice compared to uninfected mice, indicating their potential as prospective biomarkers for the diagnosis or prognosis of Staphylococcus aureus endophthalmitis.^[40]^

Exosomes are expected to become new biomarkers for eye diseases owing to their relative stability and specificity in the serum and aqueous humor of patients. However, low concentrations of exosomes in body fluids may lead to a false-negative diagnosis if used as a biomarker.^[41]^ Precise regulation of exosomes in disease progression and the ocular microenvironment may potentially address this issue.

## 5. Limitation

We acknowledge several potential limitations that may have influenced the results of this bibliometric study. First, although a rigorous and well-structured research process was employed, only publications available in the Web of Science database were selected for analysis. Google Scholar and Scopus were not searched, which could have resulted in the omission of relevant papers. Second, this study produced a language bias as it exclusively focused on English-language literature. While English remains the predominant language for academic publications worldwide, some articles have been published in non-English languages, such as Chinese and Japanese. Third, since citations of a publication usually peak from 1 to 3 years after the publication, there is a possibility that the most recent influential papers or emerging trends may have been missed. Nevertheless, we believe that our study provides meaningful insights into the current state and general trends in this field.

## 6. Conclusion

This study represents the first comprehensive bibliometric and visual analysis of EVs in eye diseases. Using CiteSpace for visual analytics, research in this area has progressed significantly over the past 20 years, with exosomes being the most widely studied EVs. China and the United States are leading countries in this field; however, there is still room for improvement in terms of cooperation and communication among various countries and institutions. The current focus of research on EVs in eye diseases is the neuroprotective effects of stem cell-derived EVs and biomarkers of eye diseases. Overall, this study provides valuable scientific insights into the role of EVs in eye diseases. In summary, this study provides a scientific perspective on the study of EVs in eye diseases and presents helpful information for researchers, funding agencies, and policymakers.

## Author contributions

**Conceptualization:** Xianke Luo, Xiaoling Yan, Jian Zhou.

**Data curation:** Dan Yin, Jian Zhou.

**Formal analysis:** Yanting Xia.

**Methodology:** Xianke Luo, Shimeng Li.

**Resources:** Xianke Luo, Dan Yin.

**Software:** Xianke Luo, Miaoran Gao, Changlu Yang.

**Supervision:** Jian Zhou.

**Validation:** Jian Zhou.

**Visualization:** Xianke Luo, Suisui Shi.

**Writing – original draft:** Xianke Luo.

**Writing – review & editing:** Jian Zhou.
